# Effect of a clinical evidence technology on patient skin disease outcomes in primary care: a cluster-randomized controlled trial

**DOI:** 10.5195/jmla.2019.581

**Published:** 2019-04-01

**Authors:** Marianne Burke, Benjamin Littenberg

**Affiliations:** Dana Medical Library, University of Vermont, Burlington, VT 05405, Marianne.Burke@uvm.edu; General Internal Medicine Research, Larner College of Medicine, University of Vermont, Burlington, VT 05405 Benjamin.Littenberg@med.uvm.edu

## Abstract

**Objective:**

Providers’ use of clinical evidence technologies (CETs) improves their diagnosis and treatment decisions. Despite these benefits, few studies have evaluated the impact of CETs on patient outcomes. The investigators evaluated the effect of one CET, VisualDx, on skin problem outcomes in primary care.

**Methods:**

A cluster-randomized controlled pragmatic trial was conducted in outpatient clinics at an academic medical center in the northeastern United States. Participants were primary care providers (PCPs) and their adult patients seen for skin problems. The intervention was VisualDx, as used by PCPs. Outcomes were patient-reported time from index clinic visit to problem resolution, and the number of follow-up visits to any provider for the same problem. PCPs who were randomly assigned to the intervention agreed to use VisualDx as their primary evidence source for skin problems. Control group PCPs agreed not to use VisualDx. Investigators collected outcome data from patients by phone at thirty-day intervals. Cox proportional hazards models assessed time to resolution. Wilcoxon-rank sum tests and logistic regression compared the need for return appointments.

**Results:**

Thirty-two PCPs and 433 patients participated. In proportional hazards modelling adjusted for provider clusters, the time from index visit to skin problem resolution was similar in both groups (hazard ratio=0.92; 95% confidence interval [CI]=0.70, 1.21; *p*=0.54). Patient follow-up appointments did not differ significantly between groups (odds ratio=1.26; CI=0.94, 1.70; *p*=0.29).

**Conclusion:**

This pragmatic trial tested the effectiveness of VisualDx on patient-reported skin disease outcomes in a generalizable clinical setting. There was no difference in skin problem resolution or number of follow-up visits when PCPs used VisualDx.

## INTRODUCTION

Health care providers across a spectrum of primary care and specialty domains regularly refer to clinical evidence technologies (CETs) to answer clinical questions [1]. As reported in provider survey and chart review studies, use of CETs such as PubMed/MEDLINE, journal articles, electronic texts, topic summaries, and Internet search engines has improved diagnosis and treatment decisions and avoided adverse events [2–6]. Despite these provider reports, few studies have evaluated the impact of CETs on patient-level outcomes. Patient-level outcomes include mortality, relief of symptoms, impact on activity, perceived benefit, and costs to the patient, such as length of hospital stay and lost work time [7]. The literature on patient outcomes of CET use is mixed. Only one published study has reported an improvement in patient outcomes. Researchers reviewed insurance claims from hospitals before and after subscribing to UpToDate (a source for comprehensive medical topic summaries). Results showed a modest reduction in morbidity and length of stay in hospitals after subscribing [8].

Hospital libraries and informatics centers acquire and make CETs available to the clinical community on the assumption that these resources have value for education, practice improvement, and outcomes of care. CET licenses can be expensive. Medical school libraries associated with teaching hospitals in the United States or Canada spent an average of US$2 million each in 2015 for medical research journals and clinical information resources [9]. While CETs, individually or in combination, have been evaluated for education and practice-level outcomes, they have not undergone rigorous evaluations with randomized trials for patient outcomes. A 2015 systematic review of electronic health information, including CETs, found no randomized trials with patient outcomes, such as utilization or relief from symptoms [10].

The broad nature and diverse goals of many CETs may discourage rigorous evaluation. However, skin conditions are a relatively circumscribed domain within the broad field of primary care. The clinical goal in many cases can be quantified as time-to-problem resolution. Likewise, the need for additional medical care after the index visit usually represents a suboptimal and expensive outcome that might be reduced by improved provider knowledge and decision support [11].

Skin problems account for 15% of primary care office visits in the United States [12], and 10 common dermatologic conditions (dermatitis, pyoderma, tinea, benign neoplasms, candida, dermatosis, warts, malignant neoplasm, sebaceous cyst, and acne) account for 77% of skin-related diagnoses in family practices. Likewise, many internal conditions manifest themselves on the skin, including malignancies, vascular conditions, anemia, endocrine disorders, and pregnancy. Most skin conditions first present, and are often diagnosed and managed, in primary care. Eight percent of all outpatient visits for skin problems result in referrals to dermatologists or return visits to primary care [13]. Limitations in the ability of primary care providers (PCPs) to diagnose skin rashes and lesions correctly have been noted in the literature [14, 15]. Some studies indicate that additional dermatology knowledge, training, and diagnostic support could improve practice and patient outcomes. General practitioners in the United Kingdom who used an online source for skin cancer diagnosis information increased their diagnostic accuracy and confidence, but referrals were not reduced [16]. Referrals to dermatology in a Veterans Affairs hospital that lacked a specific diagnosis were reduced by an intervention that trained PCPs [17].

VisualDx is a CET that presents images and text on a comprehensive range of skin conditions and symptoms that are local to the skin or manifestations of internal conditions [18]. Users can search by diagnosis or by patient characteristics and examination findings to generate a differential diagnosis list with images. Individuals, practices, and institutions license VisualDx to support medical education and patient care [19]. VisualDx has been shown to improve diagnostic competency in non–primary care settings. In one study, its use improved the differential diagnosis of cellulitis by emergency room physicians [20]. In a pilot study, diagnostic accuracy of dermatology residents and medical students increased after using VisualDx, as judged by a consultant dermatologist [21].

Given the prevalence and broad range of skin conditions seen in primary care, the need for PCPs’ improved knowledge and competency in skin disease, the availability of a dermatology-focused CET (i.e., VisualDx) that has been shown to affect clinical competence, and the lack of randomized clinical trials of any CET with patient-level outcomes, the authors proposed a clinical trial to evaluate use of VisualDx in primary care in the domain of skin disease with patient-level outcomes.

Our objective was to evaluate the effect of VisualDx on duration of symptoms and follow-up care for skin problems in a pragmatic randomized clinical trial in primary care. Recognizing that in typical clinical care, the correct diagnosis and therapy are often uncertain, that some problems resolve regardless of whether the management was technically correct, and that some resist even the most insightful management, we were concerned in this study with the net result of each episode of care—the patient outcomes—rather than the intermediate steps of management (i.e., diagnosis or treatment decisions).

## METHODS

### Study design, model, and setting

We designed a cluster-randomized controlled trial to evaluate the outcomes of skin problems for patients whose PCP referred to VisualDx or not (usual care). In this design, PCPs were the subjects of randomization. Patients were clustered in the arm of the provider they saw for the skin problem. The cluster design was appropriate because the intervention was directed to physicians, while the outcomes occur in individual patients [22]. With randomization, environmental and provider or subject characteristics (e.g., years in practice, insurance status, chronicity of the presenting complaint, comorbidities) were distributed at chance levels across both arms of the experiment.

**Figure 1:**
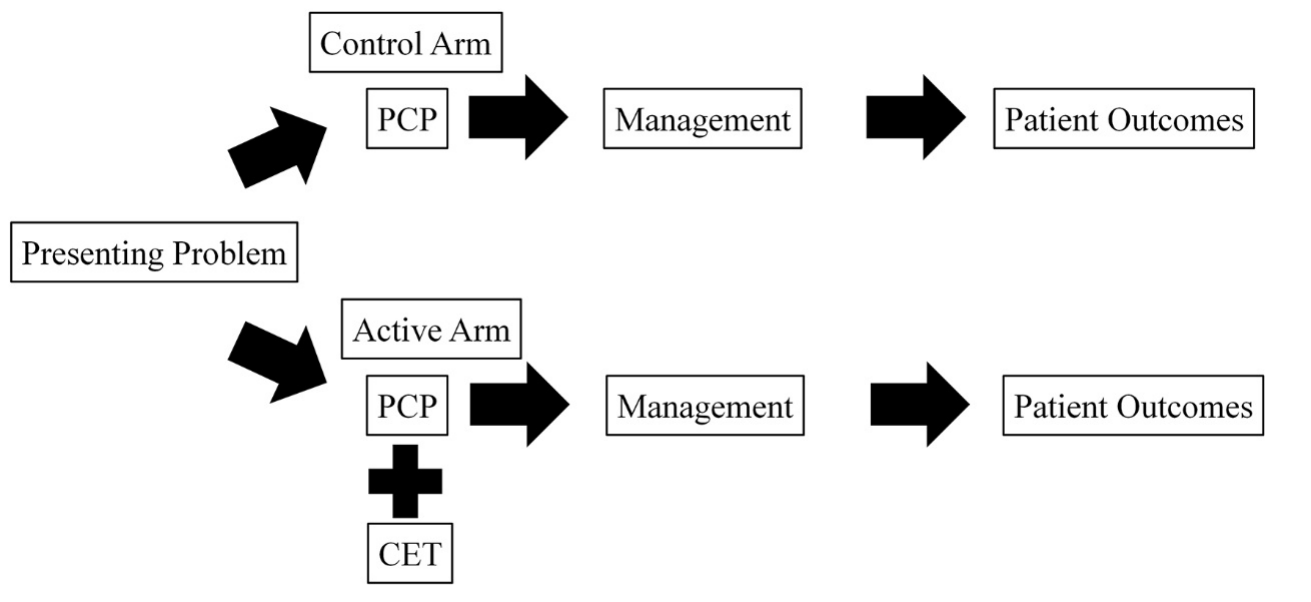
Model of the cluster-randomized pragmatic design PCP=primary care provider; CET=clinical evidence technology.

The model underlying the design of the experiment asserted that the CET supported PCPs in management (i.e., diagnosis, treatment, and referral decisions) and impacts patient-level outcomes—resolution of symptoms and return appointments—when used in a real-world clinical setting. Presumably, use of a valuable CET leads to more correct diagnoses and wiser therapeutic or referral choices. These, in turn, lead to better patient outcomes (i.e., quicker resolution of the presenting problem or reduced need for additional care). To test this model, we performed a pragmatic [23] (i.e., not heavily controlled) cluster-randomized controlled trial of the impact of one CET on the outcomes of skin problems presenting to primary care (Figure 1).

The study was conducted at clinics associated with an academic regional medical center in the northeast United States. VisualDx and other CETs were available to medical center clinicians through the hospital intranet, electronic health record, and mobile devices. The institutional review board approved the protocol in June 2015.

### Provider subjects

Attending physicians, residents, advanced practice nurses, and physician assistants in outpatient family medicine and general internal medicine were invited to participate by email or personal contact. Eligible providers (1) were currently seeing patients at a primary care site, (2) consented and agreed to comply with the protocol procedures assigned, and (3) permitted patients to be informed of the study via a letter sent over their signature. Providers answered a survey concerning resident or attending status, year of clinical degree, sex, specialty, and typical number of times per month that they used CETs for patient care (supplemental Appendix A).

We randomly assigned PCPs to intervention or control groups using a sequential numbered envelope method, stratified by resident status [24]. We randomized residents independently because of the possibility that they might respond differently to the intervention than more experienced providers would. PCPs were enrolled in the study when they gave consent, completed the tutorial, provided their signature for patient letters, and affirmed their agreement to follow their assigned protocol.

### Patient subjects

Adult patients seen for acute or chronic skin problems, excluding lacerations or burns, were eligible. Patients were excluded if they did not speak English or were decisionally impaired. To identify patients, we reviewed the appointment records of participating providers for patients who were seen for a skin problem. We identified patients with any complaint in the broad range of skin diseases as noted in the electronic health record. The reason for visit, appointment note, and clinical summary fields provided information about patient complaints, such as “rash,” “redness,” “lump,” “itch,” “wart,” “mole,” or “sore.” International Classification of Diseases codes were also used to identify potential cases. Per the institutionally approved protocol, personal health information from the patient record—such as reason for visit, phone number, and address—could be used for identification and recruitment but not to ascertain patient characteristics or outcomes.

We sent each identified patient a letter signed by their PCP describing the study and informing them that the study team would call to invite their participation. The letter also stated how to opt out of any contact.

### Intervention

The intervention was VisualDx, as used by PCPs treating patients with skin problems. Providers received email notification of their experimental group status with a link to a self-paced slide tutorial that was specific to their group (supplemental Appendixes B and C). For the “Active” group, the five-to-ten-minute tutorial included the direction to use VisualDx when needed in treating a patient skin problem and instructions on how to access and use the CET. For the “Control” group, the tutorial included the direction not to use VisualDx and a general orientation to information sources that are available through the medical library. A study team member contacted participating providers by email, phone, and letter at intervals during the study to remind them of their assigned protocol and to confirm their continued participation.

### Measurements

The primary predictor (i.e., independent variable) was the randomized group status of the provider: Active (use of VisualDx) or Control (non-use). Patient subjects were assigned to the group of the provider they saw. The primary outcome variables reported by the patients were (1) time to resolution of the skin problem from presentation at the primary care office visit and (2) number of follow up visits (to any provider) for the same problem.

About thirty days after the index visit, an investigator phoned each eligible patient (except those who had opted out) and, following verbal consent, proceeded with interview questions. If the patient reported their presenting skin problem resolved (i.e., “all better”), their participation in the study was concluded. Patients whose presenting complaint had not resolved were reinterviewed at sixty days and, if still unresolved, again at ninety days. The thirty-sixty-ninety day phone call schedule was specified in the protocol to balance the requirements to reach many people while preserving patient recall [25].

At the first interview, patients reported their ages, sexes, and whether the PCP seen was their usual provider (supplemental Appendix D). We ascertained the status of the skin problem as “all better,” “improved,” “unchanged,” or “worse” each time that we interviewed the patient. If it was “all better” at any interview, we asked them to recall the number of days from the index visit date or the date when they realized the problem was resolved. If necessary, we asked questions to aid more exact recall. This determined the “days to resolution” outcome variable. The final problem status at the last completed interview was determined for analysis.

To determine the number of follow-up appointments, at the first interview, we asked how many appointments the patient had had for the same problem since the index visit. If there was a second or third phone interview, we asked how many appointments they had had since the last call and added that number to any previously reported appointments, if any. The total number of appointments reported constituted the variable.

### Data collection

Trained research assistants using standardized scripts conducted patient interviews by phone. Study data were collected and managed using Research Electronic Data Capture (REDCap) secure tools, hosted by the researchers’ institution.

### Blinding

By necessity, providers knew their own intervention or control group status. Investigators were blind to providers’ and patients’ groups while conducting patient interviews. Patients were blind to the group assignment of their providers.

### Analysis

We used Cox proportional hazards models to assess time to resolution and Wilcoxon-rank sum tests and logistic regression to compare return appointments between groups. Logistic and proportional hazards models were adjusted for clustering. Data analyses were performed using Stata 14 statistical software. We sought an adequate sample size to detect a moderate-to-large effect of the intervention, on the order of 0.4 standard deviations. Given the broad range of skin problems presenting in primary care, we expected significant variability in the time to resolution. Therefore, we chose a target of 8 days to resolution with a standard deviation of 20 days. The effect of clustering with PCP was not known, but we used estimates from other primary care settings that suggested an intra-cluster correlation of approximately 0.025 [26]. Assuming α=0.05, β=0.80, 10 patients per provider, and a 2-sided *t*-test, we estimated the study needed 26 PCPs and 260 patients.

## RESULTS

**Figure 2:**
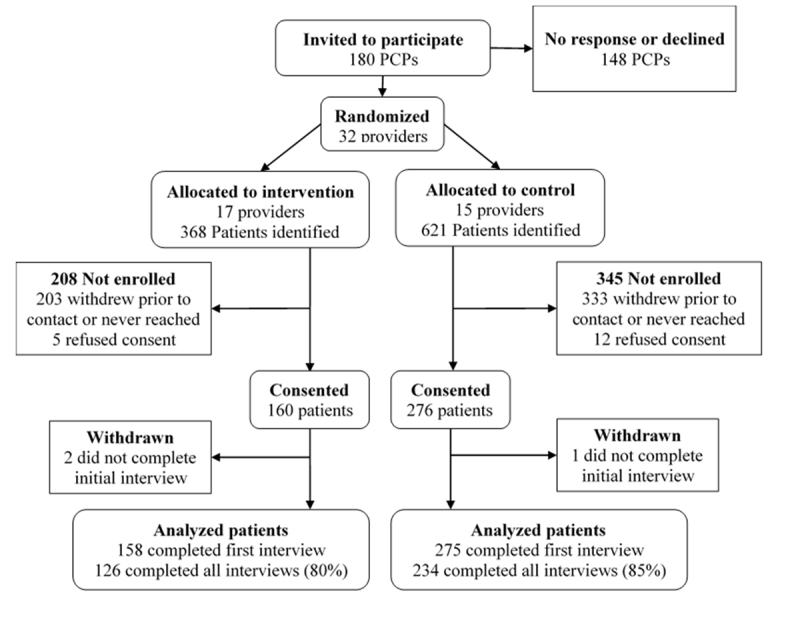
Flow of participants through stages of the randomized-cluster controlled trial

We enrolled 31 physicians and 1 nurse practitioner. We identified 989 eligible patients with a visit to a participating PCP related to a skin problem between November 2015 and August 2016. Four hundred thirty-three patients consented and provided data (Figure 2).

The Active and Control groups were similar at baseline, except for the median number of subjects per PCP (6 in the Active group versus 15 in the Control group; *p*=0.045) (Table 1). Seven PCPs (22%) reported use of VisualDx prior to the study, including 4 (27%) in the Control group who agreed not to use it during the trial.

**Table 1:**
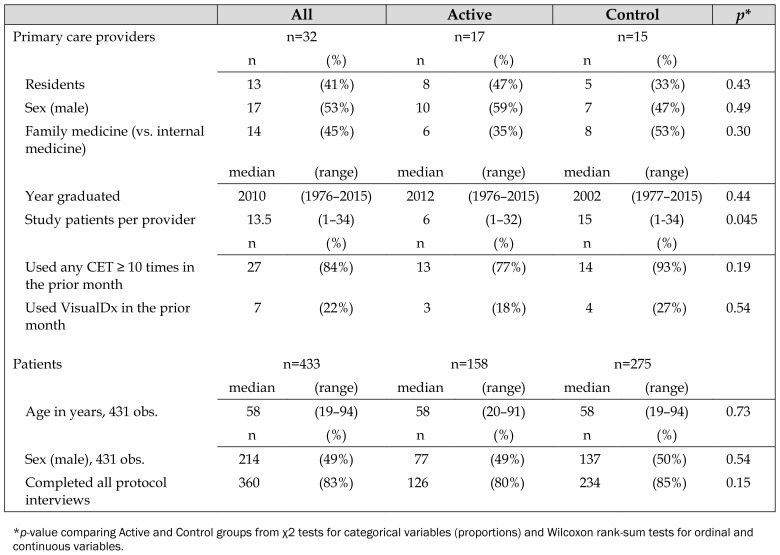
Characteristics of primary care providers and patients

	All		Active		Control		p*
Primary care providers	n=32		n=17		n=15		
	n	(%)	n	(%)	n	(%)	
	
Residents	13	(41%)	8	(47%)	5	(33%)	0.43
Sex (male)	17	(53%)	10	(59%)	7	(47%)	0.49
Family medicine (vs. internal medicine)	14	(45%)	6	(35%)	8	(53%)	0.30
	median	(range)	median	(range)	median	(range)	
	
Year graduated	2010	(1976–2015)	2012	(1976–2015)	2002	(1977–2015)	0.44
Study patients per provider	13.5	(1–34)	6	(1–32)	15	(1–34)	0.045
	n	(%)	n	(%)	n	(%)	
	
Used any CET ≥ 10 times in the prior month	27	(84%)	13	(77%)	14	(93%)	0.19
Used VisualDx in the prior month	7	(22%)	3	(18%)	4	(27%)	0.54
Patients	n=433		n=158		n=275		
	median	(range)	median	(range)	median	(range)	
	
Age in years, 431 obs.	58	(19–94)	58	(20–91)	58	(19–94)	0.73
	n	(%)	n	(%)	n	(%)	
	
Sex (male), 431 obs.	214	(49%)	77	(49%)	137	(50%)	0.54
Completed all protocol interviews	360	(83%)	126	(80%)	234	(85%)	0.15

* *p*-value comparing Active and Control groups from χ2 tests for categorical variables (proportions) and Wilcoxon rank-sum tests for ordinal and continuous variables.

### Problem resolution

**Figure 3:**
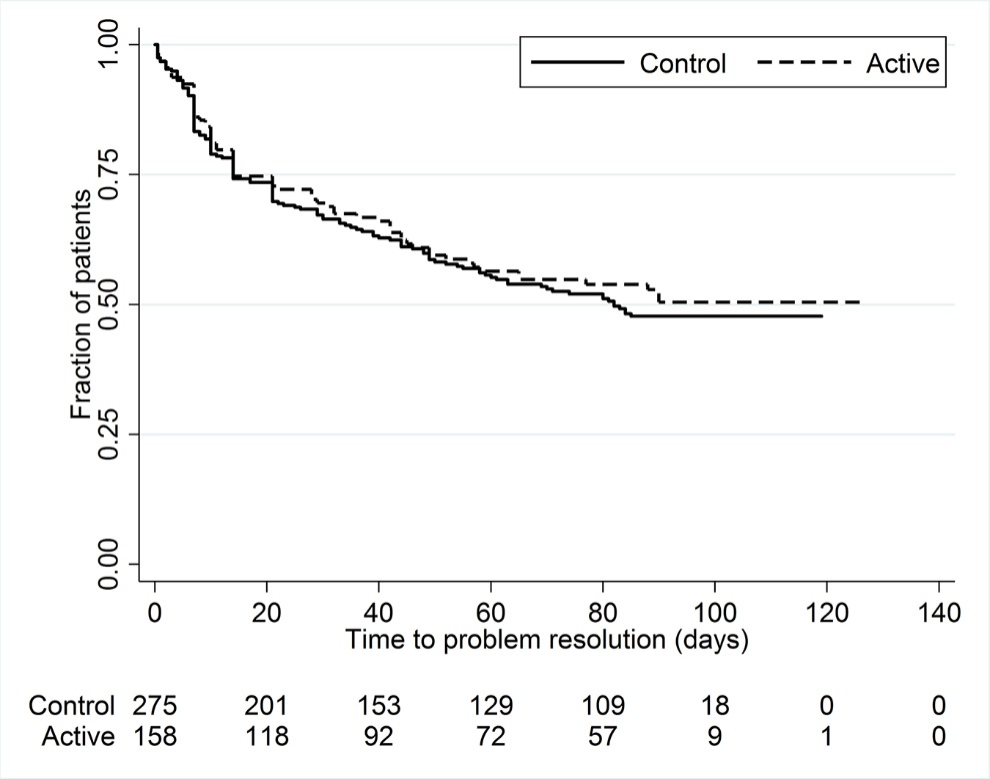
Proportion of patients whose skin problems remained unresolved over time

Nearly half (48%) of all patients in the study considered their skin problem resolved (i.e., “all better”) by the final contact, including 46% in the Active group and 49% in the Control group (*p*=0.48). Active and Control patients were similar in terms of whether they were “all better,” “improved,” “unchanged,” or “worse” at their final interview (*p*=0.88) (Table 2, Figure 3).

**Table 2:**
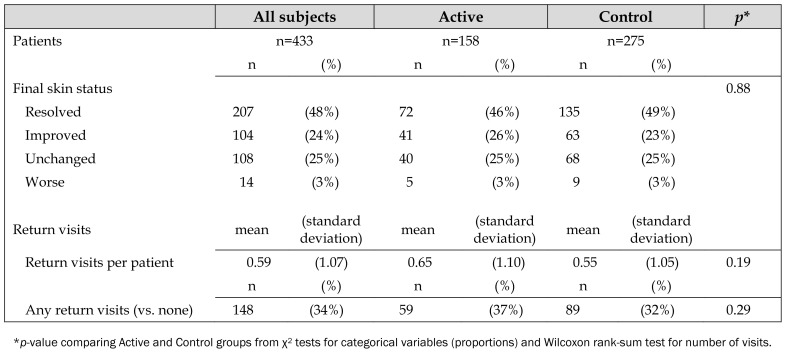
Problem resolution and return visit outcomes

	All subjects		Active		Control		p*
Patients	n=433		n=158		n=275		
	n	(%)	n	(%)	n	(%)	
	
Final skin status							0.88
Resolved	207	(48%)	72	(46%)	135	(49%)	
Improved	104	(24%)	41	(26%)	63	(23%)	
Unchanged	108	(25%)	40	(25%)	68	(25%)	
Worse	14	(3%)	5	(3%)	9	(3%)	
Return visits	mean	(standard deviation)	mean	(standard deviation)	mean	(standard deviation)	
	
Return visits per patient	0.59	(1.07)	0.65	(1.10)	0.55	(1.05)	0.19
	n	(%)	n	(%)	n	(%)	
	
Any return visits (vs. none)	148	(34%)	59	(37%)	89	(32%)	0.29

* *p*-value comparing Active and Control groups from χ^2^ tests for categorical variables (proportions) and Wilcoxon rank-sum test for number of visits.

Time to resolution was similar between groups throughout the observation period of up to 120 days (*p*=0.56 by log-rank test) (Figure 3).

**Figure 4:**
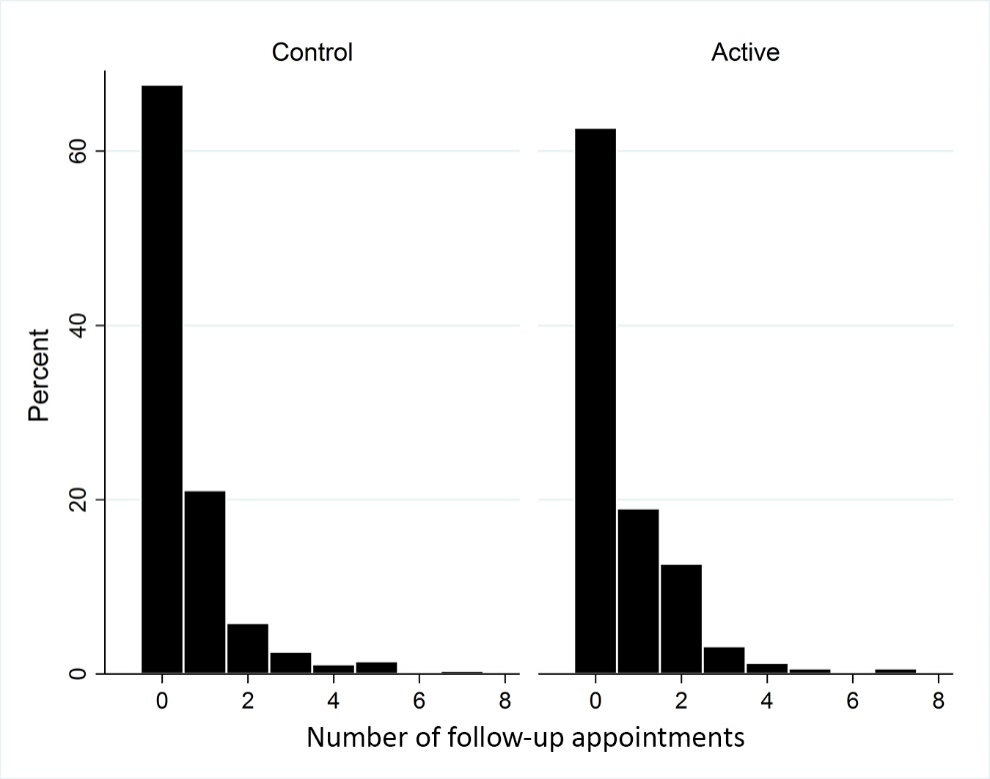
Proportion of patients whose skin problems remained unresolved over time

In univariable Cox proportional hazards models, with standard errors adjusted for provider clusters, the days from index visit to resolution were similar between groups (hazard ratio [HR]=0.92; 95% confidence interval [CI]=0.70, 1.21; *p*=0.54). Tests for potential confounding by patient age and sex, PCP status (as resident and as patient’s regular provider), PCP time since graduation, number of patients per provider, and time of the year indicated no potential confounding. Therefore, these variables were not included in the analysis.

### Return appointments

Active group patients had a mean of 0.65 return appointments compared to 0.55 in the Control group (*p*=0.19). The median was 0 return appointments in both groups (Figure 4). Thirty-seven percent of Active group patients had 1 or more follow-up appointments for the index problem, versus 32% of Control group patients (*p*=0.29).

When analyzed as a binary variable (any follow-up visits vs. none) in cluster-adjusted logistic regression, the odds of a return visit were higher in the Active group than in the Control group (odds ratio [OR]=1.25; 95% CI=0.93, 1.67; *p*=0.15), but this was not statistically significant. Tests for potential confounding by patient characteristics (age and sex), PCP characteristics (as resident, as patient’s regular provider, and time since graduation), or time of the year indicated no confounding. Therefore, these variables were not included in the model. However, the number of patients per provider was associated with the use of any follow-up visits *(p*=0.066) and group assignment (*p*=0.065), raising the possibility of confounding and warranting its inclusion in the final logistic regression model. The odds of any follow-up visits remained higher in the Active group than in the Control group, when adjusting for clustering and the number of patients per provider (OR=1.14; 95% CI=0.84, 1.56; *p*=0.39), but this was not statistically significant. The intra-cluster correlation coefficient for both outcome measures was <0.00001 with an upper 95% confidence limit of 0.039.

## DISCUSSION

Patients with skin problems whose PCPs used VisualDx experienced similar rates of problem resolution and similar time to resolution as patients whose providers did not use this CET. There was no difference in the number of follow-up visits to any health care provider for the index skin problem.

The goal of this study was to assess the effectiveness of a CET as used in a generalizable clinical setting rather than to determine its mechanism of action or efficacy under ideal conditions. Therefore, we designed a “pragmatic trial” in a clinical environment in which day-to-day factors were not highly controlled. Pragmatic trials seek to answer the question, “Does this intervention work under usual conditions?” [23]. Intervention PCPs had flexibility in how they followed their assigned protocols to reference VisualDx when uncertainty about patient care arose. They could have searched in VisualDx by diagnosis terms, as opposed to using the differential diagnosis support tool. They could also have decided that assistance was not needed with some patients and opt not to employ the CET and could seek advice from additional sources after consulting VisualDx.

We obtained data for the primary outcomes from patient reports because we sought to understand the outcomes of care as experienced by the patients. Patient-reported outcome measures complement other health care indicators such as provider-reported outcomes, chart review, and insurance data. They are appropriate measures in research when the intervention is incorporated into treatment [27, 28] and are frequently used in clinical trials of medical products, drugs, and health-related quality-of-life studies [29].

We did not evaluate whether the diagnosis or treatment that the PCP decided upon was correct by an objective standard, such as expert dermatologist review. Likewise, we did not distinguish appropriate follow-up appointments or referrals from unnecessary or avoidable ones, recording only that a follow-up occurred.

Physician-reported benefits of referring to CETs—such as correct diagnosis, treatment, and avoidance of adverse events—have been previously noted. In a multi-institutional survey of physicians (n=4,906) and residents (n=1,290) in 118 hospitals, Marshall et al. found that 36% of physicians and 42% of residents changed a diagnosis after referring to a clinical evidence source in a recalled, recent incident. Physicians (29%) and residents (32%) also reported avoiding unnecessary procedures or tests because of the information that they used in the incident [5].

Likewise, use of VisualDx may improve diagnostic skills. A team including the developer of VisualDx reported that among 28 cases initially misdiagnosed as cellulitis in the emergency room, VisualDx included the correct diagnosis in its differential diagnosis list more often than the admitting medical residents (64% vs. 14%; *p*=0.003). In a pilot study by Chou, clinical diagnoses of 13 patients were made by 13 dermatology residents and 51 medical students before and after using VisualDx. Diagnostic accuracy increased from 63% to 81% (*p*<0.01) as judged by a consultant dermatologist [21]. Despite these positive intermediate effects, the published literature, including the study reported here, provides no evidence of better patient outcomes.

Why did use of VisualDx—a technologically sophisticated, well-designed, state-of-the-art CET—fail to influence the tested outcomes for skin disease? Some potential reasons for the negative results in this trial, such as bias due to uneven distribution of patient or provider characteristics, were minimized by the randomized design of the study. Another reason we found no difference between groups could be that the VisualDx users had insufficient knowledge of the resource to use it effectively. However, Active group PCPs were made aware of the resource, what it was meant to do, and how to access it. They received more training on its features, via an online tutorial, than is usually available in clinical practice. Although the VisualDx interface appears intuitive and easy to use compared to other CETs, it is possible that PCPs had difficulty finding the information that they needed. The specific content and interactive diagnosis tool of VisualDx, written largely by specialists, could be too complex or time consuming in the primary care setting. This may have contributed to busy clinicians bypassing VisualDx at times, resulting in suboptimal management.

Even if the content acquired by the PCPs was correct from a biomedical point-of-view, the PCPs were not obligated to follow it. Indeed, local availability of certain procedures, prescriptions, and specialty referrals may make it unreasonable or impractical to follow the advice of the CET, potentially leading to the “no difference” result.

Finally, it is possible that many skin problems presenting in primary care are inherently resistant to improvement no matter how well managed. They will resolve (or not) at their own pace regardless of the diagnosis and therapy offered. Nonetheless, return appointments and referrals to dermatology could conceivably be reduced with optimal primary care management.

This study tested the effectiveness of VisualDx for problem resolution and return visit frequency, not for other outcomes such as improved diagnosis or satisfaction with care. This was not a comprehensive multi-attribute assessment of the CET. Likewise, ease of use and usefulness were also beyond the scope of this evaluation.

As VisualDx is costly, this study may help health care organizations determine whether that cost is appropriate for their local institutional goals and settings.

### Strengths and limitations

The randomized-cluster parallel design reduced the likelihood of bias due to differences in provider and patient subjects. Secular events occurring outside the study, such as seasonal changes in skin-related appointments, affected providers and patients in the intervention and control groups equally because of the randomized, parallel design.

The study took place in one large academic medical center, possibly reducing generalizability to other settings. However, the patients of the study institution are similar to populations in rural regions of the United States in terms of age, race, poverty rates, and other factors.

Although this was the largest randomized study of a CET with patient outcomes to date, the power to detect a potential effect was limited. Given the sample size of 433 patients, a control resolution rate of 49% within 90 days, and assumption of α=0.05, the study had 80% power to detect a resolution rate of at least 63% in the Active group, using χ^2^ analysis. The observed rate was 46% and, therefore, not significantly different from Control. In the Cox model, the observed HR of 0.92 (favoring Control) was well under the minimum detectable HR of 1.24. Likewise, the study had 80% power to detect a difference of 0.30 return visits per patient. The observed rate was 0.10 higher in the Active group. Given that all analyses showed a trend toward worse outcomes (i.e., longer time to resolution and more return visits in the Active group), it is highly unlikely that a larger study would have demonstrated a statistically significant beneficial effect.

The study relied on provider adherence to the protocol based on their agreement to do so (which was confirmed periodically). We did not have independent confirmation of their adherence. There might also have been contamination between provider subjects since there were both Active and Control providers in some clinics. While Active group PCPs used VisualDx as their primary resource for skin-related uncertainty and Control group PCPs did not, both groups could use other CETs and resources that were available in the information-rich environment of the academic medical center. This access could have masked a positive effect of using VisualDx.

We had limited ability to independently measure participant usage of VisualDx prior to the study. However, at baseline, 22% of PCPs reported use of VisualDx in the prior month with no significant difference between groups. We did not measure VisualDx use during the study. Nevertheless, we did encourage provider adherence to the protocol. When contacted, all providers confirmed that they were staying within their assigned protocol of using or not using VisualDx as a reference.

The study relied upon the memory of patients, which could have been faulty. However, the first patient interviews followed the index visit by approximately thirty days, which is a relatively short time span [25]. Only one patient who consented could not remember the skin problem visit at all.

This study included patients with acute and chronic conditions reflecting the usual variety of skin conditions that are seen in primary care. It is possible that a study of only acute skin conditions or a study in an inpatient setting would have had a different outcome.

### Implications

While VisualDx did not make a difference in the patient outcomes studied, it may have value for other goals such as medical knowledge, decision confirmation, and diagnostic confidence. The pragmatic study design with patient-level outcomes proved to be feasible and could be extended to evaluate other clinical evidence source technologies that are relevant to health care.

## CONCLUSION

The study showed no difference in resolution of symptoms and return visits in patients of doctors who referenced VisualDx. Although VisualDx and other CETs can support institutional missions of medical knowledge and practice improvement, VisualDx does not appear to improve patient outcomes for skin problems managed in primary care.

## SUPPLEMENTAL FILES

Appendix A Provider eligibility and baseline survey

Appendix B Primary care providers Active group educational tutorial

Appendix C Primary care providers Control group educational tutorial

Appendix D Patient interview data collection instruments
