# Supplementary material for: Effect of a clinical evidence technology on patient skin disease outcomes in primary care: a cluster-randomized controlled trial
Source: J Med Libr Assoc. 2019 Apr 1;107(2):151–62. doi: 10.5195/jmla.2019.581 (PMC6466492; doi:10.5195/jmla.2019.581)
Supplement: Appendix A [file jmla-107-151-s001.pdf]

JMLA Journal of the Medical Library Association

Supplemental content to
J Med Libr Assoc. Apr;107(2):dx.doi.org/10.5195/jmla.2019.581
www.jmla.mlanet.org
© Burke, Littenberg 2019

Effect of a clinical evidence technology on patient skin disease outcomes in primary care: a cluster-randomized controlled trial

Marianne Burke, MA, AHIP; Benjamin Littenberg, MD

APPENDIX A

Provider eligibility and baseline survey

This survey was administered to primary care providers through REDCap following their consent to participate.

Eligibility screening questions:

1. What is your medical or professional degree?
   - Physician
   - Physician assistant
   - Advanced practice nurse
   - None of the above

2. Are you currently seeing patients at a University of Vermont Medical Center Family Medicine or Primary Care Internal Medicine Clinic?
   - Yes
   - No

3. Do you agree to adhere to the procedures of the study, depending on the group you are randomized to?
   - Yes
   - No
   - Not sure. Please call me.

Answers to Q1. must be physician, physician assistant, or advanced practice nurse. Answer to Q2. must be yes. Answer to Q3. must be yes to be enrolled in the study and continue to baseline survey.

Baseline survey in the information for skin problems in primary care study

1. In the last month, how often did you refer to a print or electronic information source to support patient care?
   - None at all
   - 1–3
   - 4–6
   - 7–9
   - 10 or more times

JMLA Journal of the Medical Library Association

SC

Supplemental content to
J Med Libr Assoc. Apr;107(2):dx.doi.org/10.5195/jmla.2019.581
www.jmla.mlanet.org
© Burke, Littenberg 2019

2. Which information sources did you use to support patient care? (Check all that apply.)
   - o DynaMed
   - o Google
   - o Journal articles
   - o PubMed/MEDLINE
   - o UpToDate
   - o Textbooks (electronic or print)
   - o VisualDx
   - o Not sure/don't remember
   - o Other

3. If other, please specify.

The next three questions refer to when you saw patients for skin problems.

4. In the last month, how many times did you see a patient for a skin problem?
   - o None at all
   - o 1–3
   - o 4–6
   - o 7–9
   - o 10 or more times

5. In the last month, how many times did you look for additional information to support care for a patient skin problem?
   - o None at all
   - o 1–3
   - o 4–6
   - o 7–9
   - o 10 or more times

6. Recalling those times when you sought information for a patient skin problem, what sources did you use? (Select all that apply.)
   - o DynaMed
   - o Google
   - o Journal articles
   - o PubMed/MEDLINE
   - o UpToDate
   - o Textbooks (electronic or print)
   - o VisualDx
   - o Not sure/don't remember
   - o Other

6A. If other please specify: ______________________________

JMLA Journal of the Medical Library Association

Supplemental content to
J Med Libr Assoc. Apr;107(2):dx.doi.org/10.5195/jmla.2019.581
www.jmla.mlanet.org
© Burke, Littenberg 2019

Information about you

7. What year did you graduate from professional school? ____________________

8. Are you in a residency program?
   - o Yes
   - o No

9. Primary care specialty
   - o Family medicine
   - o Internal medicine
   - o Other

9A. If other please specify: ____________________

10. Your usual practice location:
   - o Family Medicine Berlin
   - o Family Medicine Colchester
   - o Family Medicine Hinesburg
   - o Family Medicine Milton
   - o Family Medicine South Burlington
   - o Urgent Care Colchester
   - o Adult Primary Care South Burlington
   - o Adult Primary Care Essex
   - o Adult Primary Care Burlington
   - o Adult Primary Care Williston

11. What is your gender?
   - o Female
   - o Male
   - o Other

12. Please let us know here if you have any questions or comments.

We will email you in a few days with an orientation for the procedures for your randomized group.

[End of provider baseline survey]
