# Supplementary material for: Effect of a clinical evidence technology on patient skin disease outcomes in primary care: a cluster-randomized controlled trial
Source: J Med Libr Assoc. 2019 Apr 1;107(2):151–62. doi: 10.5195/jmla.2019.581 (PMC6466492; doi:10.5195/jmla.2019.581)
Supplement: Appendix B [file jmla-107-151-s002.pdf]

JMLA Journal of the Medical Library Association

Supplemental content to
J Med Libr Assoc. Apr;107(2):dx.doi.org/10.5195/jmla.2019.581
www.jmla.mlanet.org
© Burke, Littenberg 2019

Effect of a clinical evidence technology on patient skin disease outcomes in primary care: a cluster-randomized controlled trial

Marianne Burke, MA, AHIP; Benjamin Littenberg, MD

APPENDIX B

Primary care providers Active group educational tutorial

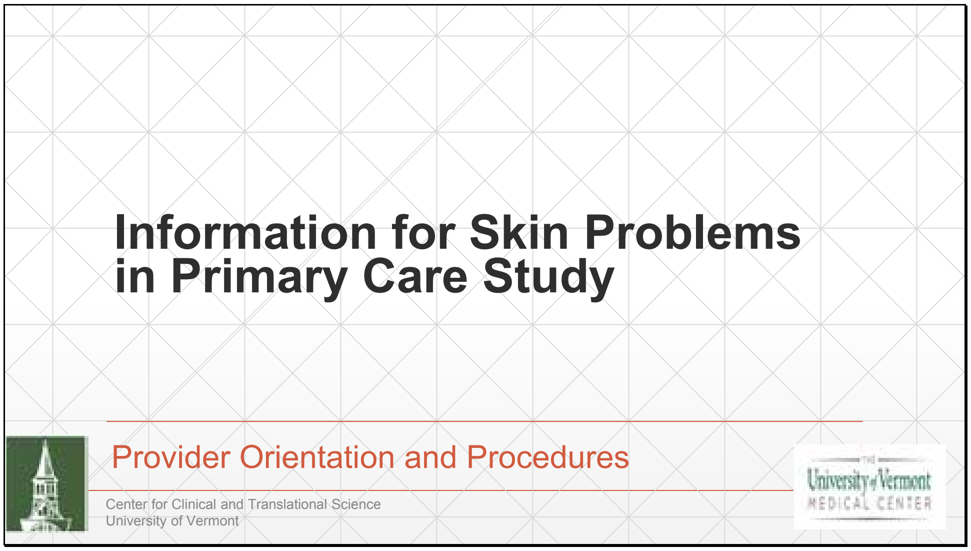

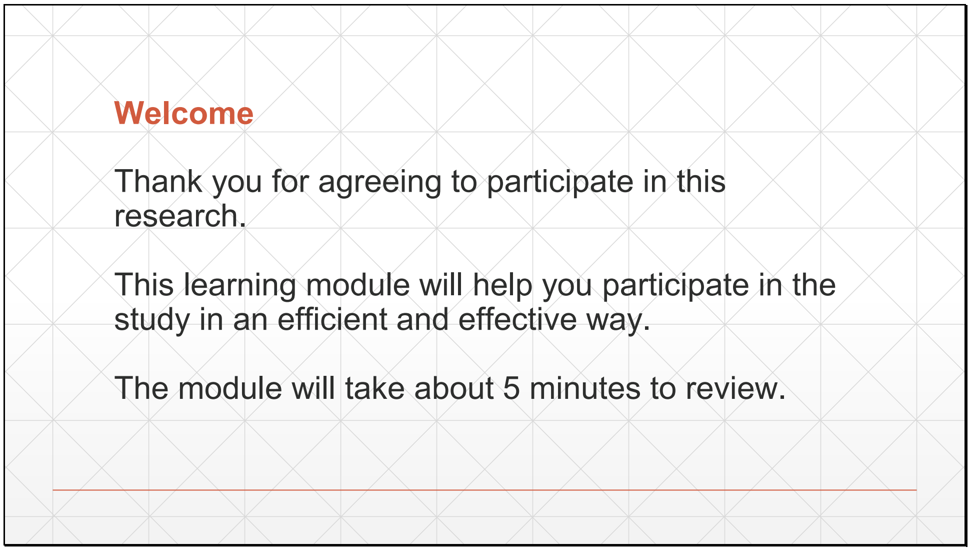

Your Group Protocol

- Your group protocol is to refer to **VisualDx,** a dermatology information source, when you see a patient for a skin problem.
- If you have any uncertainty about appearance, diagnosis, treatment, or prognosis of a skin problem or want to share information with a patient, please use **VisualDx**.

Your Group Protocol

- The referral to VisualDx related to the patient skin problem could be anytime before, during, or after a visit,
- The lookup could be brief to confirm something you already know or to share an image or description with your patient.
- The lookup would be longer if you have more concern about the diagnosis or treatment of the problem.

What Is VisualDx?

- VisualDx is a clinical knowledge and diagnostic support resource that is licensed by UVM Medical Library to support providers and patient care.
- It contains over 100,000 images of skin and other visible conditions.

How Do I Access VisualDx?

There are 4 main ways:

- PRISM
- UVMMC Intranet Desktop
- Dana Medical Library website
- Your Mobile Device (app)

The next few slides will provide instruction on each access method.

From the PRISM Home Screen

- Click the blue **EPIC** button at the top left of PRISM screen for a dropdown menu
- Click on **Reference Links** for another dropdown menu
- Click on **VisualDx**

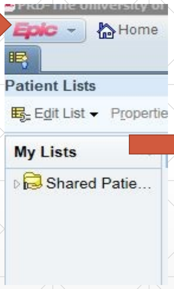

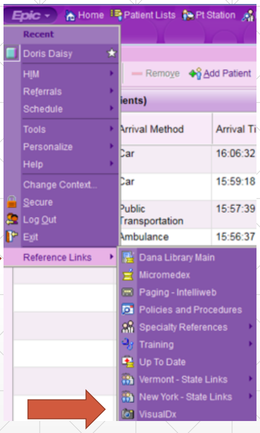

From the UVMMC Intranet

- On the UVMMC intranet home page
- Look for the **Applications** box and click the **General** button
- Click on **Dana Medical Library**
- **This will bring you to the Dana Library website**

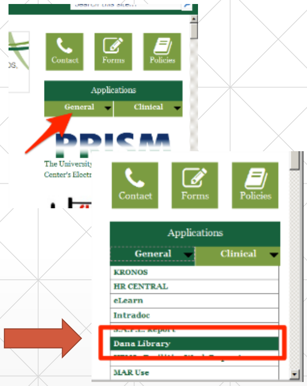

From the Dana Library Website

- Click the **Articles and Database** link
- Scroll down to the **Clinical Databases section** in the left hand column
- Click the **VisualDx** link
- Bookmark the page for convenience.
- Your most direct link to VisualDx is through PRISM or Mobile app.

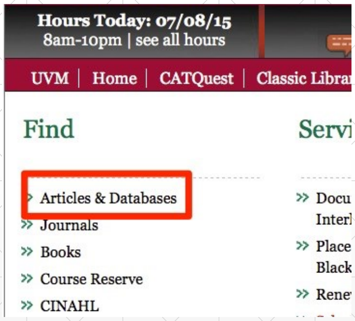

To Access VisualDx from your Mobile Device

- Go to PRISM or Dana Library website
- Go to VisualDx
- Directions for registering and downloading the app are on the opening page. Click on the icon.
- Video: http://www.visualdx.com/video-tutorials/visualdx-mobile

Get our free app on your iPhone, iPad, or Android device.

The next two slides introduce ways to use VisualDx.

How to use VisualDx: Differential Builder

- The differential builder is a distinguishing feature of VisualDx. Click on the blue Differential Builder button to start.
- The builder will prompt you for patient age, lesion type, body location, appearance, and other findings.
- VisualDx will display images and diagnoses that match all the criteria you selected.

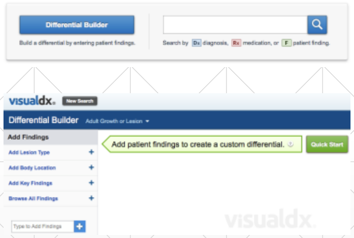

VisualDx Features

- Please take time to become familiar and fluent with using VisualDx.
- For more information, see the educational videos at the VisualDx website: http://www.visualdx.com/visualdx-videos/5-minute-overview-and-demo
- If you have any problem using VisualDx easily, contact Gary Atwood MLS, Study Team member and Education Librarian at Dana Medical Library at Gary.Atwood@uvm.edu or 656-4488.

Other Procedures: What Happens After the Patient Visit?

- PI identifies your eligible patients in PRISM.
- Study team sends each patient a letter over your name informing them of the study.
- We ask that you provide your signature to include on the letters to your patients.

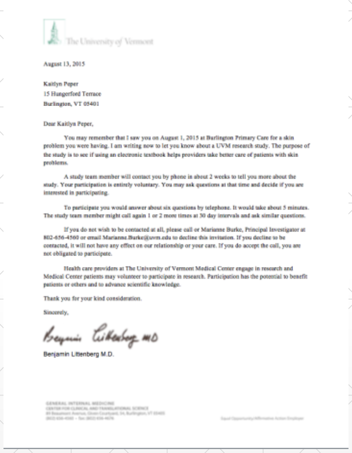
The University of Vermont

August 13, 2015

Kaitlyn Peper
15 Hungerford Terrace
Burlington, VT 05401

Dear Kaitlyn Peper,

You may remember that I saw you on August 1, 2015 at Burlington Primary Care for a skin problem you were having. I am writing now to let you know about a UVM research study. The purpose of the study is to see if using an electronic textbook helps providers take better care of patients with skin problems.

A study team member will contact you by phone in about 2 weeks to tell you more about the study. Your participation is entirely voluntary. You may ask questions at that time and decide if you are interested in participating.

To participate you would answer about six questions by telephone. It would take about 5 minutes. The study team member might call again 1 or 2 more times at 30 day intervals and ask similar questions.

If you do not wish to be contacted at all, please call or Marianne Burke, Principal Investigator at 802-656-4560 or email Marianne.Burke@uvm.edu to decline this invitation. If you decline to be contacted, it will not have any effect on our relationship or your care. If you do accept the call, you are not obligated to participate.

Health care providers at The University of Vermont Medical Center engage in research and Medical Center patients may volunteer to participate in research. Participation has the potential to benefit patients or others and to advance scientific knowledge.

Thank you for your kind consideration.

Sincerely,

Benjamin Littenberg MD

Benjamin Littenberg M.D.

Additional Information about the PCP Role

- Both PCPs and patients are subjects in this study.
- 30 or more providers and 300 patients, an average of 10 patients per provider, will participate.
- We will contact you every 2 weeks by email or phone to update you on the progress of the study and answer any questions.
- We will notify you when we have recruited enough patients so that you no longer need to follow the protocol.
- Anticipated duration of your involvement is 6 – 12 weeks.

Questions or concerns?

If you have any problem using VisualDx easily, contact Gary Atwood MLS, Study Team member and Education Librarian at Dana Medical Library at Gary.Atwood@uvm.edu or 656-4488.

If you have a concern about the study or its procedures, please contact the Principal Investigator, Marianne Burke, MA-L, at the Center for Clinical and Translational Science, 4th fl. Given Courtyard South, UVM, by email mburke@uvm.edu or phone 802-236-0075.

This research protocol was approved by the UVM/UVMMC Committee on Human Subjects Research, June 10, 2015.

Thank you for completing this module. We recommend you download it for your reference. Please return to the REDCap survey page to answer 1 question.

Center for Clinical and Translational Science
University of Vermont
656- 4560

THE University of Vermont MEDICAL CENTER
