# Supplementary material for: Effect of a clinical evidence technology on patient skin disease outcomes in primary care: a cluster-randomized controlled trial
Source: J Med Libr Assoc. 2019 Apr 1;107(2):151–62. doi: 10.5195/jmla.2019.581 (PMC6466492; doi:10.5195/jmla.2019.581)
Supplement: Appendix C [file jmla-107-151-s003.pdf]

JMLA Journal of the Medical Library Association

SC

Supplemental content to
J Med Libr Assoc. Apr;107(2):dx.doi.org/10.5195/jmla.2019.581
www.jmla.mlanet.org
© Burke, Littenberg 2019

Effect of a clinical evidence technology on patient skin disease outcomes in primary care: a cluster-randomized controlled trial

Marianne Burke, MA, AHIP; Benjamin Littenberg, MD

APPENDIX C

Primary care providers Control group educational tutorial

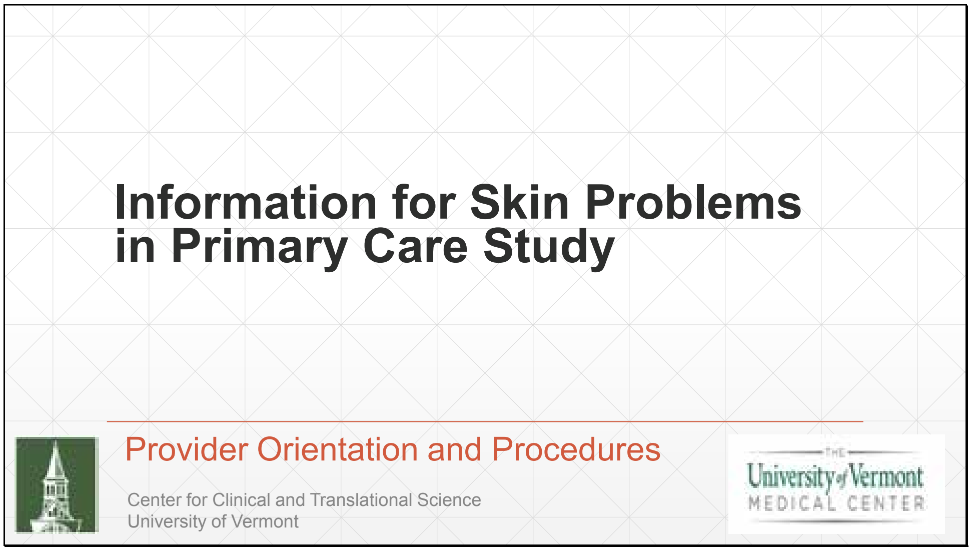

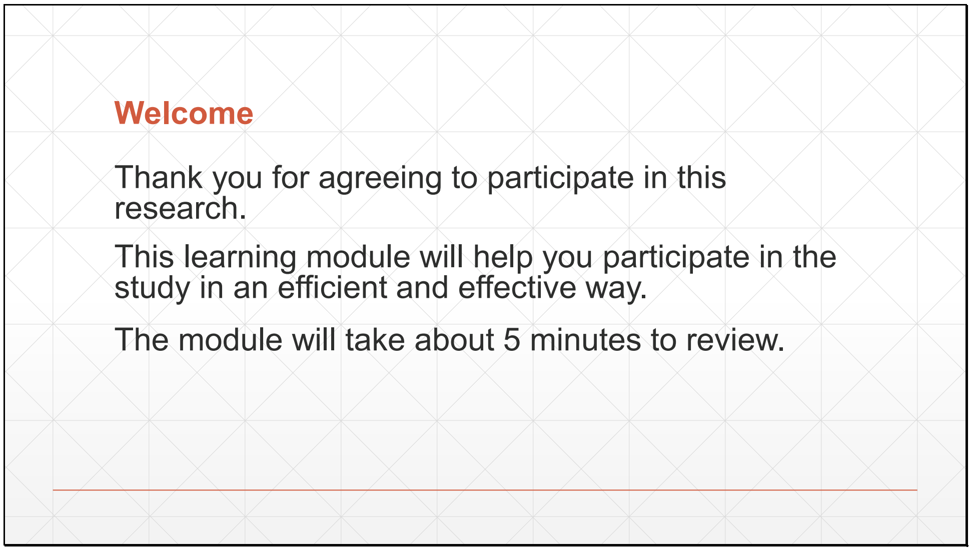

Your Group Protocol

- You will see patients for skin problems as you normally do.
- Note the skin problem discussed or treated during the visit in the PRISM patient record as you normally would.

Your Group Protocol

- According to your group protocol, you may choose to refer to an additional information source concerning the skin problem <u>or not.</u>
- Refer to any textbook or electronic resource as you normally would or not.
- <u>Except</u> – Do **not** refer to **VisualDx.**

Other Procedures: What Happens After the Patient Visit?

- PI identifies your eligible patients in PRISM
- Study team sends each patient a letter over your name informing them of the study.
- We ask you to provide your signature to include on the letters to your patients.

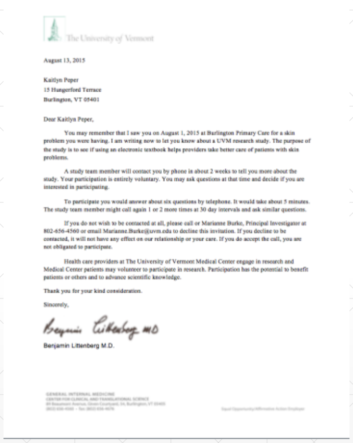

The University of Vermont

August 13, 2015

Kaitlyn Peper
15 Hungerford Terrace
Burlington, VT 05401

Dear Kaitlyn Peper,

You may remember that I saw you on August 1, 2015 at Burlington Primary Care for a skin problem you were having. I am writing now to let you know about a UVM research study. The purpose of the study is to see if using an electronic textbook helps providers take better care of patients with skin problems.

A study team member will contact you by phone in about 2 weeks to tell you more about the study. Your participation is entirely voluntary. You may ask questions at that time and decide if you are interested in participating.

To participate you would answer about six questions by telephone. It would take about 5 minutes. The study team member might call again 1 or 2 more times at 30 day intervals and ask similar questions.

If you do not wish to be contacted at all, please call or Marianne Burke, Principal Investigator at 802-656-4560 or email Marianne.Burke@uvm.edu to decline this invitation. If you decline to be contacted, it will not have any effect on our relationship or your care. If you do accept the call, you are not obligated to participate.

Health care providers at The University of Vermont Medical Center engage in research and Medical Center patients may volunteer to participate in research. Participation has the potential to benefit patients or others and to advance scientific knowledge.

Thank you for your kind consideration.

Sincerely,

Benjamin Littenberg M.D.

GENERAL INTERNAL MEDICINE
CENTER FOR CLINICAL AND TRANSLATIONAL SCIENCE
[illegible]

Additional Information about the PCP Role

- Both PCPs and patients are subjects in this study.
- 30 or more providers and 300 patients, an average of 10 patients per provider, will participate.
- We will contact you every 2 weeks by email or phone to update you on the progress of the study and answer any questions.
- We will notify you when we have recruited enough patients so that you no longer need to follow the protocol.
- Anticipated duration of your involvement is 6 - 12 weeks.

Access to Clinical Knowledge Resources

Dana Medical Library provides access to electronic clinical knowledge resources to support evidence-based patient care:

- Medical research journals.
- Medical e-text books in primary care and specialties.
- PubMed database with links to full text journal articles
- Mobile clinical apps.

You can access these resources from many different locations.

FYI: How to Access Clinical Information Sources

There are 3 main ways:

- PRISM
- UVMMC Intranet Desktop
- Dana Medical Library website

The next few slides will provide instruction on each access method

Access From PRISM

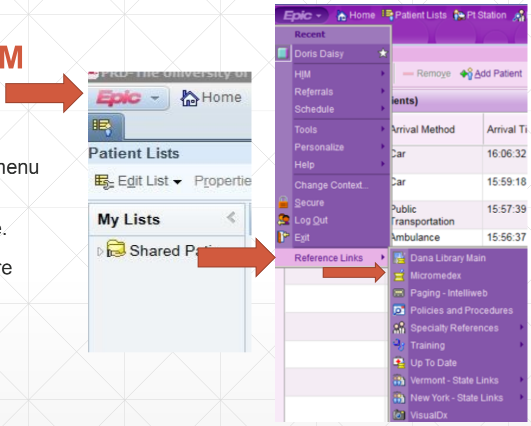

- Click the **EPIC** button
- Click on **Reference Links** for menu
- Click on **Dana Library Main** to connect to the library's web site.
- **UpToDate** and **Micromedex** are also available here.
- **Do not use VisualDX.**

From the UVMMC Intranet

- Log in with M number
- On the UVMMC intranet home page
- http://intranet.fletcherallen.org
- Look for the **Applications** box and click the **General** button
- Click on **Dana Medical Library**
- **This will bring you to the Dana Library website**

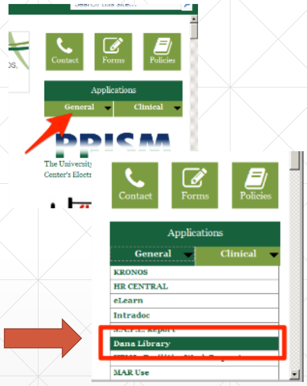

Access from the Dana Medical Library Web Site http://library.uvm.edu/dana

- Click on PubMed etc.
- OR Click the **Articles and Databases** link.
- Scroll down to the Clinical Databases section in the left hand column.
- Find multiple clinical information resources.
- **From Off Campus:** Log on using M number and password.

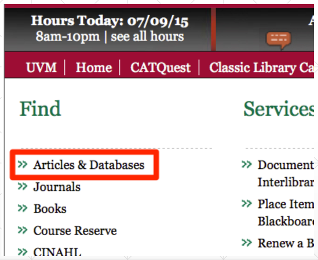

Questions or concerns?

If you have any problems referring to library resources easily, contact Gary Atwood MLS, Study Team member and Education Librarian at Dana Medical Library at Gary.Atwood@uvm.edu or 802-656-4488.

If you have a concern with any aspect of the study protocol please contact the Principal Investigator, Marianne Burke at mburke@uvm.edu or phone 802-236-0075.

This research protocol was approved by the UVM/UVMMC Committee on Human Subjects Research, June 10, 2015.

Thank you for completing this module. We recommend you download it for your reference. Please return to the REDCap survey page to answer 1 question.

Center for Clinical and Translational Science
University of Vermont
656-4560

The University of Vermont MEDICAL CENTER
