# Supplementary material for: Effect of a clinical evidence technology on patient skin disease outcomes in primary care: a cluster-randomized controlled trial
Source: J Med Libr Assoc. 2019 Apr 1;107(2):151–62. doi: 10.5195/jmla.2019.581 (PMC6466492; doi:10.5195/jmla.2019.581)
Supplement: Appendix D [file jmla-107-151-s004.pdf]

JMLA Journal of the Medical Library Association

SC

Supplemental content to
J Med Libr Assoc. Apr;107(2):dx.doi.org/10.5195/jmla.2019.581
www.jmla.mlanet.org
© Burke, Littenberg 2019

Effect of a clinical evidence technology on patient skin disease outcomes in primary care: a cluster-randomized controlled trial

Marianne Burke, MA, AHIP; Benjamin Littenberg, MD

APPENDIX D
Patient interview data collection instruments

Patient telephone interview/questionnaire at ~30 days after index visit for the skin problem

Study team investigators, including trained research assistants, reached identified eligible patients by telephone thirty days or after the index visit for the skin problem. After review of the lay summary and verbal consent from the patient, the following interview was conducted.

1. Our records show that you saw Dr. [provider name] for a skin problem about [date of visit]. Does that sound right?
   - Yes – Go to question 2
   - No – If patient cannot recall the problem or the visit, call is ended and patient is ineligible [dis-enrolled].

2. Is Dr. [provider name] your usual primary care doctor?
   - Yes
   - No

3. Since you saw Dr. [provider name] for the skin problem last month have you gone back to him/her or to any other doctor for a follow-up appointment for this same problem?
   - Yes – Go to question 4
   - No – Go to question 6

   3A. How many other appointments did you have? ______________________________

   3B. What kind of doctor did you see for the follow up appointment?
   - Primary care
   - Dermatologist
   - Other

   3C. If other, what kind? ______________________________

4. Since that visit with Dr. [provider name], would you say the skin problem is:
   - All better – If all better Go to 4A–4D
   - Improved – Go to 5
   - Unchanged – Go to 5
   - Worse – Go to 5

   4A. About how long after that visit did it take to be all better? ______________________

   4B. How many days or weeks after the appointment was it all better? __________________

   4C. Would you say that this time frame is exact or approximate?
   - exact
   - approximate

Journal of the Medical Library Association
Supplemental content to
J Med Libr Assoc. Apr;107(2):dx.doi.org/10.5195/jmla.2019.581
www.jmla.mlanet.org
© Burke, Littenberg 2019

4D. If it's easier, thinking back from today, how many days or weeks ago did you think the problem was all better? [Answer could be in days, weeks, or a date.]

____

After patient answers question 4A, or 4B, or 4C with enough clarity to determine number of days – Go to Question 6.

5. Do you plan to see a provider [of any kind] again for this same skin problem?
- o Yes – Go to 5A
- o No – Go to 6

5A. What type of doctor will you see?
- o Primary care
- o Dermatologist
- o Other

5B. If other, what type? ______________________________

6. Thank you. Now, I'd like to ask a couple of questions about you.
How old are you? ______________________________

7. What is your sex?
- o male
- o female
- o other

After question 7, Go to End call 1 or End call 2 depending on problem status.

End call 1: Patients who were "All Better"
Thank you for helping with this research. I have all I need and won't need to call you again.
Do you have any questions or comments?
[Record if any] ______________________________
Thank you for your participation. Goodbye.

End call 2: Patients who were improved unchanged or worse.
We're coming to the end of questions today. I'll call you again in about 4 weeks to see if anything about the skin problem has changed. Thank you for your participation.

Sixty day or second patient telephone interview

1. Hello [patient's first name] [patient's last name]. This is [caller name] from the University of Vermont Medical Center Medical Center research on skin problems. We spoke with you a month ago about a skin problem you saw Dr. [provider name] for. Do you remember talking with us?
- o Yes
- o No

2. When we spoke with you before, you described the problem as [problem status: improved, unchanged, or worse]. Does that sound right?
- o Yes
- o No

JMLA Journal of the Medical Library Association

SC

Supplemental content to
J Med Libr Assoc. Apr;107(2):dx.doi.org/10.5195/jmla.2019.581
www.jmla.mlanet.org
© Burke, Littenberg 2019

3. Since I [or team member name] spoke with you before on [date of call] about the skin problem visit, have you gone back to Dr. [provider name] or any other provider for this same problem?

- o Yes – Go to 3A and 3B
- o No – Go to 4

3A. Since we spoke with you last time, how many appointments have you had for this same problem?

____

3B. What type of doctor did you see for this appointment(s)?

- o Primary care
- o Dermatologist
- o Other

If other, what type?

____

4. Since that [first] visit with Dr. [provider name] and our conversation last month would you say the condition is:

- o All Better – If all better, go to 4A, 4B, or 4C, 4D
- o Improved – Go to 5
- o Unchanged – Go to 5
- o Worse – Go to 5

4A. When did you realize the skin problem was all better? [Look at calendar try to approximate days after call.]

____

4B. Looking at a calendar, that was about [day/date] about _____ [days time] after we talked

____

4C. If it's easier, thinking back from today, can you recall how long ago you realized the problem was all better? When was that?

____

4D. Would you say that number of days is exact or approximate?

- o Exact number of days
- o Approximate number of days

After determining the days since index visit or date [all better], Go to End Call 1

5. Do you plan to see a doctor or health care provider again for this condition?

- o Yes – Go to 5A
- o No – Go to End Call 2

5A. What type of doctor are you planning to see next?

- o Primary care
- o Dermatologist
- o Other

If other, what type? ______________________________

Go to End Call 2

End Call 1: Thank you for helping with this research.

JMLA Journal of the Medical Library Association

Supplemental content to
J Med Libr Assoc. Apr;107(2):dx.doi.org/10.5195/jmla.2019.581
www.jmla.mlanet.org
© Burke, Littenberg 2019

I have all I need and won't need to call you again. Do you have any questions or comments?

End Call 2: We're coming to the end of questions today. I'll call you again in about 4 weeks to see if anything about the skin problem has changed.

Ninety day (final) patient questionnaire

Hello [patient's first name] I'm calling from the UVM Medical Center about the skin problems study. I [or one of my team members] called you about [3 or 4] weeks ago with questions about what happened with a skin problem you saw Dr. [provider name] for.

1. Do you remember?
   - o Yes
   - o No

2. When we spoke with you before said the problem was...[INTERVIEWER SAYS WHICH] [improved, unchanged, or worse]. Does that sound right?
   - o Yes
   - o No

3. Since I [or my team member] spoke with you before [4 weeks ago] [date of call] about your skin problem visit, have you gone back to any other doctor or provider for this same problem?
   - o Yes – Go to 3A–C
   - o No – Go to 4

   3A. How many follow up appointments?

   ____

   3B. What type of doctor did you see for this appointment(s)?
   - o Primary care
   - o Dermatologist
   - o Other

   3C. If other, what type? ______________________________

4. I'm going to ask you how the skin problem is doing.

Since that first visit with Dr. [provider name] and our conversation last month would you say the condition is:

   - o All Better – Go to 4A–4D
   - o Improved – Go to 5
   - o Unchanged – Go to 5
   - o Worse – Go to 5

   4A. So about when did you realize the skin problem was all better? ______________________________

   4B. Looking at a calendar, that was about [suggest day/date]. That was about _____ [time] after we talked.

   ____

Journal of the Medical Library Association
Supplemental content to
J Med Libr Assoc. Apr;107(2):dx.doi.org/10.5195/jmla.2019.581
www.jmla.mlanet.org
© Burke, Littenberg 2019

4C. If it's easier, thinking back from today, can you recall how long ago you realized the problem was all better? When was that?

____

4D. Would you say that number of days is exact or approximate?

- o exact
- o approximate

Go to 5 End Call

5. End Call [all]:

We have come to the end of the call and your participation in the study. Thank you so much for your help. Do you have any questions or comments before we end the call?

[Record comments if any] ________________________________

Again, thank you very much. Goodbye
